# Effects of An Eight-Week Resistance Training on Plasma Vaspin Concentrations, Metabolic Parameters Levels and Physical Fitness in Patients with Type 2 Diabetes

**Published:** 2014-10-04

**Authors:** Hassan Amouzad Mahdirejei, Sajedeh Fadaei Reyhan Abadei, Arshin Abbaspour Seidi, Negar Eshaghei Gorji, Hassan Rahmani Kafshgari, Mostafa Ebrahim Pour, Habib Bagheri Khalili, Farshad Hajeizad, Mohamad Khayeri

**Affiliations:** 1 Department Of Exercise Physiology, Faculty of Physical Education and Sport Science, Islamic Azad University Sari Branch, Sari, Iran; 2 Physical Education and Sport Science Faculty, Shomal Non-Governmental University, Amol, Iran; 3 Department of Exercise Physiology, Faculty of Physical Education and Sport Science, University of Payam Noor Behshahr Branch, Behshahr, Iran

**Keywords:** Resistance Training, Vaspin, Lipid, Type 2 Diabetes

## Abstract

Vaspin as a novel adipokine has insulin-sensitizing effects, which may be associated with decreased blood glucose concentration. In this study, we aimed to investigate the effects of resistance exercise training on plasma vaspin concentrations and its relation to plasma levels of insulin and glucose in patients with type 2 diabetes (T2D). In a quasi-experimental study, 18 male patients with T2D (mean age, 48.50 ± 7.73 years, mean weight, 79.41 ± 12.60 kg) were divided into 2 groups as follows: control (n=9), and resistance training (RT; n=9) groups. Resistance training was performed 3 times weekly for 8 weeks. Anthropometric, metabolic parameters and plasma vaspin levels were measured at baseline and at the end of study. Within-group data were analyzed with the paired t test, and between-group effects were analyzed with the independent t test. Waist-hip ratio (WHR), glucose, insulin of plasma and insulin resistance [homeostasis model assessment of insulin resistance (HOMA-IR) score] were all significantly decreased, whereas levels of vaspin and plasma lipids [cholesterol, triglycerides (TG), high-density lipoprotein (HDL), low-density lipoprotein (LDL) and very low-density lipoproteins (VLDL)] showed no significant changes in RT group as compared with the related values of control groups. Serum vaspin levels did not correlate with anthropometric and metabolic parameters at the assigned times. Our findings suggest that 8-week of resistance training significantly improved insulin resistance index; however, this form of exercise failed to result in significant changes in serum vaspin concentration and lipid profiles. Further research is needed to investigate the role of vaspin in human physiology and to elucidate the effect(s) of exercise intervention on serum vaspin concentrations (Registration Number: IRCT2013060911772N1).

Clinical studies have shown that loss of muscle mass in individuals with type 2 diabetes (T2D) increases the risk of developing glucose intolerance (1). Also, patients with T2D often experience increases in adipose tissue (2). On the other hand, adipose tissue is a metabolically endocrine organ that secretes adipocytokines (3).

Vaspin identified as a visceral adipose tissuederived serine protease inhibitor is a novel adipocytokine that has been suggested as a compensatory factor against the insulin resistance state of metabolic syndrome (3). It has been suggested that vaspin levels are related to systemic insulin resistance, indicating possible contribution to the pathogenesis of diabetes (4). Youn et al. (4) reported that vaspin levels are higher in individuals with impaired insulin sensitivity. Oberbach et al. (5) observed a significant reduction in serum vaspin concentrations after an hour acute exercise bout as well as after 4 weeks of training, concluding exercise-induced oxidative stress reduces vaspin serum concentrations, but this type of exercise fails to make any improvement in insulin sensitivity. However, the effects of insulin resistance and lipid profile changes, induced by resistance training (RT) exercise, on plasma vaspin concentrations in patients with T2D are yet unclear. Thus, the aim of the present study was to investigate the effects of RT exercise on vaspin and lipid profile levels and to evaluate the possible relationship between metabolic parameters and plasma vaspin concentrations in patients with T2D.

Eighteen adult men with T2D participated in this quasi-experimental study. Subjects were assigned to RT group (mean age, 47.60 ± 7.7 years; mean weight=79.5 ± 15.4 kg, n=9) and control group (mean age, 49.62 ± 8.05 years, mean weight=79.3 ± 8.8, n=9). This study was approved by the Ethics Committee of Islamic Azad University of Sari Branch. Furthermore, all participants signed an informed consent form. Subjects were excluded if they had a known history of stroke or transient ischemic attack, uncontrolled hypertension, severe dyslipidemia, acute or chronic inflammatory disease, or any other serious diseases. To reduce drug effects, we selected subjects with glycated hemoglobin values under 9% who received over 1,000 mg of metformin per day, and drug dosages were maintained throughout the study. Before initiating the tests, the participants underwent familiarization sessions and participated in one-repetition maximum (1-RM) test. To estimate the 1-RM, three days prior to the experiment, participants underwent 1-RM test for the following exercises: bench press, butterfly, lat pull-down, biceps curl, triceps extension, seated rowing, knee flexion, knee extension, and heel raise (6).

The RT groups participated in an 8-week (on 3 nonconsecutive days per week) of supervised circuit resistance exercise program. The programs were composed of three following steps: warm-up for 10-15 minutes, circuit resistance exercise for 45-60 minutes, and cool down for 10 minutes. Resistance exercise program consisting of 10 isotonic exercises with 50- 80% of 1-RM were performed in a circuit. During the .rst and fourth weeks of training, the resistance was set at 50-70% of each individual’s 1-RM with 8-15 repetitions of each exercise movement within 45-60 seconds. Thereafter, the goal was to achieve between 70-80% of the current 1-RM with 8-10 repetitions of each exercise movement within 45-60 seconds. The participants performed 3 circuits of 10 exercises per session of training. The intervals between each exercise were 30-60 seconds and between each circuit was 120-180 seconds. Ten exercises were used for training (7, 8). Blood samples were collected in the morning after the 12-hour overnight fast, before and after an 8-week of exercise program. Plasma vaspin levels were determined with vaspin enzyme-linked immunosorbent assay (ELISA) kit (Cusabio Biotech, Wuhan, China). Serum triglyceride (TG), total cholesterol (TC), high density lipoprotein (HDL), and very low density lipoprotein (VLDL) were measured by an enzymatic photometric analyzer (Pars Azmoun, Tehran, Iran), while low density lipoprotein (LDL) was determined using a modified version of the Friedewald formula (FF) (9). Plasma glucose was determined using glucose oxidase-peroxidase/4- aminoantipyrine (GOD-PAP) method (Pars Azmoun, Tehran, Iran). Serum insulin concentrations were determined by ELISA kit (Mercodia, Uppsala, Sweden). Hemoglobin A1C (HbA1C) levels were measured by enzymatic and chromatographic methods using commercial kits (Bio-system S.A., Germany). Insulin resistance was determined by calculating the homeostasis model assessment of insulin resistance (HOMA-IR) score using the following formula (8):

$$\mathrm{HOMA-IR}=\frac{\left[\mathrm{Insulin (U/l)}\times \mathrm{Fasting blood glucose (mmol/l)}\right]}{22.5}$$

All data were expressed as mean ± standard deviations (SD) and were analyzed using Statistical Package for the Social Sciences (SPSS, SPSS Inc., Chicago, IL, USA) version 15.0. The differences between two groups were examined by an independent samples t test, while the before and the after comparisons within group were performed using paired t-tests. The relation between variables was assessed using Pearson’s method. A p value under 0.05 was considered statistically significant in the interpretation of the results.

Metabolic parameters, vaspin levels, anthropometric and muscle strength performance characteristics of all participants are demonstrated in tables 1 and 2, at baseline and at the end of study. There are no significant differences between two groups regarding anthropometric characteristics, muscle strength performance, metabolic parameters (TC, TG, HDL, LDL, VLDL, and HbA1C), and vaspin levels (Tables1, 2). Since glucose, insulin and HOMA-IR levels decreased significantly, there are significant differences regarding these values between two groups.

Also, our findings confirmed that there is no correlation between vaspin levels with insulin sensitivity, glucose tolerance and lipid profiles in preand post-exercise, shown in table 3.

**Table 1:** Anthropometric and muscle strength performance characteristics before and after 8 weeks of training programs


Variables	Group	Baseline	8 weeks	P value

Age (Y)	RT	47.60 ± 7.7	-	-
	Control	49.62 ± 8.05	-	
Anthropometric measurement				
Height (cm)	RT	169.19 ± 7.95	-	-
	Control	172.81 ± 6.45	-	
Weight (kg)	RT	79.5 ± 15.4	80.4 ± 14.4	0.93
	Control	79.3 ± 8.8	80.3 ± 9.1	
BMI (kg/m^2^)	RT	28.0 ± 4.9	26.9 ± 4.5	0.38
	Control	26.3 ± 3.7	26.4 ± 3.7	
BFP (%)	RT	22.1 ± 3.0	23.1 ± 3.2	0.27
	Control	24.5 ± 4.4	23.3 ± 4.6	
WHR	RT	0.91 ± 0.22	0.86 ± 0.22 *	0.05 **
	Control	0.75 ± 0.09	0.75 ± 0.09	
VO_2_max ( ml/kg/min)	RT	26.16 ± 1.15	32.08 ± 1.41 *	0.04 **
	Control	29.53 ± 1.17	27.40 ± 1.39	
Muscle strength performance				
1-RM bench press (kg)	RT	51.4 ± 8.4	84.4 ± 10.7 *	0.00 **
	Control	49.48 ± 27.7	36.7 ± 3.9	
1-RM knee extension (kg)	RT	64.4 ± 9.5	85.3 ± 12.5 *	0.00 **
	Control	51.4 ± 14.9	56.1 ± 6.8	
1-RM lat pull-down (kg)	RT	73.4 ± 16.6	94.1 ± 13.2 *	0.00 **
	Control	42.9 ± 4.5	45.6 ± 3.8 *	
1-RM triceps extension (kg)	RT	64.4 ± 9.5	85.3 ± 12.5 *	0.04 **
	Control	51.4 ± 14.9	56.1 ± 6.8	
1-RM heel raise (kg)	RT	132.3 ± 33.3	179.3 ± 32.4 *	0.00 **
	Control	115.3 ± 13.9	121.1 ± 10.7 *	
1-RM arm curl (kg)	RT	59.3 ± 5	85.3 ± 12.5 *	0.10
	Control	60 ± 1.51	71.5 ± 20.9	
1-RM butterfly (kg)	RT	65.5 ± 9.4	92.8 ± 6.5 *	0.00 **
	Control	56.9 ± 3.2	58.6 ± 2.8	
1-RM knee flexion (kg)	RT	53.6 ± 10.2	91.5 ± 9.6 *	0.00 **
	Control	58.6 ± 2.2	58.3 ± 6.1	
1-RM crunch	RT	26.5 ± 12.1	43.4 ± 8.6 *	0.00 **
	Control	21 ± 3.9	23.3 ± 6.2	
1-RM seated rowing (kg)	RT	108 ± 14	133.3 ± 10.5 *	0.10
	Control	79 ± 19.9	93.3 ± 6.2 *	


The values are presented as mean ± standard deviation (SD). *; Significant differences within groups at (p≤0.05) show, **; Significantly differences between groups (p ≤ 0.05) show, BMI; Body mass index, BFP; Body fat percentage, WHR; Waist-hip ratio and 1-RM; One-repetition maximum.

**Table 2:** Metabolic parameters and vaspin levels before and after 8 weeks of training programs


Variables	Group	Baseline	8 weeks	P value

Vaspin (ng/ml)	RT	319.50 ± 75.75	283.40 ± 133.67	0.380
	Control	353.75 ± 31.90	391 ± 194.72	
TC (mg/dL)	RT	180 ± 55.51	171.10 ± 37.91	0.72
	Control	176.50 ± 31.78	160.87 ± 28.33	
TG (mg/dL)	RT	175.80 ± 72.44	174.9 ± 84.60	0.08
	Control	196.75 ± 75.24	132.87 ± 44.99	
HDL-C (mg/dL)	RT	39.20 ± 9.65	42.80 ± 8.87 *	0.72
	Control	40.25 ± 12.63	41.93 ± 8.21	
LDL-C (mg/dL)	RT	83.40 ± 26.95	88.60 ± 28.72	0.39
	Control	92 ± 23.07	106.50 ± 22.36	
VLDL-C (mg/dL)	RT	35.16 ± 14.48	34.98 ± 16.92	0.08
	Control	39.35 ± 15.04	26.57 ± 8.99	
FBS (mg/dL)	RT	187.40 ± 42.06	164.40 ± 48.26	0.040 **
	Control	153.25 ± 24.24	159.12 ± 27.22	
Insulin (μU/mL)	RT	16.77 ± 13.14	8.12 ± 3.78 *	0.021 **
	Control	17.78 ± 5.77	20.62 ± 12.67	
HOMA-IR	RT	7.77 ± 6.43	3.32 ± 1.87 *	0.011 **
	Control	6.93 ± 3.16	8.15 ± 5.37	
HbA1C (%)	RT	8.33 ±1.73	8 ± 1.25	0.07
	Control	7.58 ± 1.51	8.6 ± 1.35	


**Table 3:** Pearson’s correlation coefficients between plasma vaspin concentration and other parameters at baseline and end of programs


Variables	Baseline		After 8 weeks	
	r	P value	r	P value

Body weight (kg)	0.903	0.31	0.301	0.225
BMI (kg/m^2^)	0.482	0.177	0.135	0.594
BFP (%)	0.397	0.213	0.197	0.319
WHR	0.630	0.122	0.111	0.660
TC (mg/dL)	0.328	0.2444	0.451	0.190
TG (mg/dL)	0.611	0.128	0.344	0.237
HDL-C (mg/dL)	0.140	0.362	0.394	0.214
LDL-C (mg/dL)	0.070	0.437	0.102	0.397
VLDL-C (mg/dL)	0.611	0.128	0.241	0.291
FBS (mg/dl)	0.331	0.243	0.197	0.433
Insulin (mU.L)	0.235	0.295	0.253	0.310
HOMA-IR	0.455	0.188	0.268	0.282
HbA1C	0.732	0.903	0.345	0.161


In the current study, we found a significant improvement in insulin resistance without any changes in vaspin and lipid profile concentration. Vaspin circulating levels are likely to reflect its expression in the adipose tissue, while there are several reporters shown vaspin is sex- dependent and its level is related to body mass index (BMI), to parameters of insulin sensitivity, and to glucose metabolism in humans (3). Kloting et al. (10) reported that vaspin mRNA expression is higher in diabetic patients than in NGT individuals. However, no difference was found in serum vaspin level between diabetic patients and normal glucose tolerance (NGT) subjects in other studies (4, 11). Youn et al. (4) reported that physical training for 4 weeks in untrained individuals (men and women) causes increased serum vaspin concentrations with weight loss. On the other hand, Oberbach et al. (5) reported a significant reduction in serum vaspin concentrations after one hour acute aerobic exercise bout in healthy young men, concluding exercise- induced oxidative stress reduces vaspin serum concentrations. However, the result from the current study is in agreement with recent studies in which they found no changes in vaspin concentration after lifestyle modification in adults (12). To our knowledge, only Oberbach et al. (5) has investigated response of vaspin after an acute bout of exercise. It seems that the differences between the results found in the present study and those reported by Oberbach et al. (5) may be related to the type of exercise (resistance vs. endurance), suggesting that vaspin serum concentrations are decreased by exercise-induced oxidative stress, confirmed by present study. Also, it seems that it could be due to difference in age of participants of these studies. Different growth stages (ages) could affect levels of growth hormone (GH). In a study by Gonzalez et al. (13), they have shown that circulating GH concentrations increased vaspin circulating levels and its expression. Knowing that most of our subjects are elderly men with large variation in GH levels, this could explain the large variation of vaspin levels between two groups.

In agreement with previous studies about middle- aged patients with T2D (14, 15), in this study, our training program induced a marked increase in HDL levels in RT group without any significant modification in other variables of the lipid profile. RT is recommended by the American College of Sports Medicine (16) and the American Diabetes Association (17) as an effective tool to prevent and to treat metabolic diseases. Low HDL and high TG levels have been reported in males with T2D (18, 19). Also, T2D patients with serum TG above 2.3 mmol/l have a 2-fold increased risk of coronary heart disease (CHD) (19). In addition, low levels of physical activity and cardiorespiratory fitness are independent risk factors of CHD in the general population. Improvement in physical fitness has been shown to induce health benefits in term of morbidity, mortality and improvement in CHD risk factors such as visceral adipose tissue and hypertriglyceridemia (20). There is also a 2-year study showing a slight increase in HDL-cholesterol levels with exercise, but its mechanism is yet unknown (21). Those studies showing increased HDL generally involved more rigorous training regimens (22, 23), although there is some disagreement on this point as well (24). To consider the fact that our study demonstrated an increase in HDL level in the treatment group, our findings indicates that our subjects may exercise at high intensity. Although there is some suggestion that men with low HDL levels are less likely to respond to training than men with higher HDL levels (25), our data support this concept.

In the current study, we found a significant improvement in insulin resistance without any changes in vaspin concentration. Insulin resistance means an impaired biological response to insulin by one or more of its target tissues, leading to a reduction in glucose disposal rate (8). Relationship between exercise training with prevention and management of T2D has a long history (26). Earlier studies suggested that resistance training, similar to endurance training, could improve insulin resistance as well (8, 26). It is reported that physical activity increases the amount of GLUT4 which is associated with glucose uptake in skeletal muscle tissue (26). The results of the present study show that after resistance exercise, plasma levels of glucose and insulin significantly decreased. In the study conducted by Jurimae et al. (27), they observed no changes in insulin concentrations after 6,000 m rowing ergometer test in highly trained male rowers, while glucose levels were significantly increased after the exercise and decreased after the first 30 minutes of recovery. In another study, Jamurtas et al. (28) reported that after 45 minutes of sub-maximal aerobic exercise with 65% of maximal oxygen consumption, overweight males showed an increase in insulin sensitivity and a significant decrease in insulin concentration, only immediately after exercise. There is considerable inter-individual variability in the metabolic response to resistance exercise and the concomitant changes in glucose and insulin dynamics (29, 30).

In conclusion, this study showed that RT significantly decreased levels of glucose, insulin and HOMA-IR in elderly patients with T2D. However, this form of exercise failed to result in significant changes in serum vaspin concentration and lipid profiles. We found no association between changes in serum vaspin concentrations and changes in plasma levels of insulin and glucose to confirm the possible role of vaspin as a regulator of glucose metabolism. Further studies are needed to investigate the role of vaspin in human physiology and to elucidate the contradictory results regarding the effect(s) of exercise intervention on serum vaspin concentration.

The current study has some limitations. The study population was relatively small; however, the study had sufficient power to detect the influence of RT complications on serum vaspin levels.

Furthermore, a group of men were only included in our study, so we were not able to extrapolate the conclusions to the entire population. Finally, short exercise intervention with no long term follow-up, no specific diet, and no changes in other lifestyle associated physical activity were included in our exercise training period.

## References

[1] Strasser B, Siebert U, Schobersberger W (2010). Resistance training in the treatment of the metabolic syndrome: a systematic review andmeta-analysis of the effect of resistance training on metabolic clustering in patients with abnormal glucosemetabolism. Sports Med.

[2] Zoico E, Di Francesco V, Mazzali G, Vettor R, Fantin F, Bissoli L (2004). Adipocytokines, fat distribution, and insulin resistance in elderly men and women. J Gerontol A Biol Sci Med Sci.

[3] Gulcelik NE, Karakaya J, Gedik A, Usman A, Gurlek A (2009). Serum vaspin levels in type 2 diabetic women in relation to microvascular complications. Eur J Endocrinol.

[4] Youn BS, Klöting N, Kratzsch J, Lee N, Park JW, Song ES (2008). Serum vaspin concentrations in human obesity and type 2 diabetes. Diabetes.

[5] Oberbach A, Kirsch K, Lehmann S, Schlichting N, Fasshauer M, Zarse K (2010). Serum vaspin concentrations are decreased after exercise-induced oxidative stress. Obes Facts.

[6] Mazzeo RS, Tanaka H (2001). Exercise prescription for the elderly: current recommendations. Sports Med.

[7] Dunstan DW, Daly RM, Owen N, Jolley D, De Courten M, Shaw J (2002). High-intensity resistence traning improves glycemic control in older patients with type 2 diabetes. Diabetes Care.

[8] Colberg SR, Sigal RJ, Fernhall B, Regensteiner JG, Blissmer BJ, Rubin RR (2010). Exercise and type 2 diabetes: The American College of Sports Medicine and the American Diabetes Association: joint position statement. Diabetes Care.

[9] Levy JC, Matthews DR, Hermans MP (1998). Correct homeostasis model assessment (HOMA) evaluation uses the computer program. Diabetes Care.

[10] Kloting N, Berndt J, Kralisch S, Kovacs P, Fasshauer M, Schön MR (2006). Vaspin gene expression in human adipose tissue: association with obesity and type 2 diabetes. Biochem Biophys Res Commun.

[11] Gulcelik NE, Karakaya J, Gedik A, Usman A, Gurlek A (2009). Serum vaspin levels in type 2 diabetic women in relation to microvascular complications. Eur J Endocrinol.

[12] Kim JY, Kim ES, Jeon JY, Jekal Y (2011). Improved insulin resistance, adiponectin and liver enzymes without change in plasma vaspin level after 12 weeks of exercise training among obese male adolescents. Korean J Obes.

[13] González CR, Caminos JE, Vázquez MJ, Garcés MF, Cepeda LA, Angel A (2009). Regulation of visceral adipose tissue-derived serine protease inhibitor by nutritional status, metformin, gender and pituitary factors in rat white adipose tissue. J Physiol.

[14] 1Maiorana A, O'Driscoll G, Goodman C, Taylor R, Green D (2002). Combined aerobic and resistance exercise improves glycemic control and .tness in type 2 diabetes. Diabetes Res Clin Pract.

[15] Dunstan DW, Puddey IB, Beilin LJ, Burke V, Morton AR, Stanton KG (1998). Effects of a short-term circuit weight training program on glycaemic control in NIDDM. Diabetes Res Clin Pract.

[16] Donnelly JE, Blair SN, Jakicic JM, Manore MM, Rankin JW, Smith BK (2009). American College of Sports Medicine Position Stand.Appropriate physical activity intervention strategies for weight loss and prevention of weight regain for adults. Med Sci Sports Exerc.

[17] Sigal RJ, Kenny GP, Wasserman DH, Castaneda-Sceppa C, White RD (2006). Physical activity/exercise and type 2 diabetes: a consensus statement from the American Diabetes Association. Diabetes Care.

[18] Barrett-Connor E (1992). Lower endogenous androgen levels and dyslipidemia in men with non-insulin-dependent diabetes mellitus. Ann Intern Med.

[19] Laakso M, Lehto S, Penttilä I, Pyörälä K (1993). Lipids and lipoproteins predicting coronary heart disease mortality and morbidity in patients with non-insulin-dependent diabetes. Circulation.

[20] Ross R, Dagnone D, Jones PJ, Smith H, Paddags A, Hudson R (2000). Reduction in obesity and related comorbid conditions after diet-induced weight loss or exerciseinduced weight loss in men.A randomized, controlled trial. Ann Intern Med.

[21] Katzmarzyk PT, Leon AS, Rankinen T, Gagnon J, Skinner JS, Wilmore JH (2001). Changes in blood lipids consequent to aerobic exercise training related to changes in body fatness and aerobic fitness. Metabolism.

[22] King AC, Haskell WL, Young DR, Oka RK, Stefanick ML (1995). Long-term effects of varying intensities and formats of physical activity on participation rates, fitness, and lipoproteins in men and women aged 50 to 65 years. Circulation.

[23] Byrne HK, Wilmore JH (2001). The effects of a 20-week exercise training program on resting metabolic rate in previously sedentary, moderately obese women. Int J Sport Nutr Exerc Metab.

[24] Smutok MA, Reece C, Kokkinos PF, Farmer C, Dawson P, Shulman R (1993). Aerobic versus strength training for risk factor intervention in middle-aged men at high risk for coronary heart disease. Metabolism.

[25] Williams PT, Stefanick ML, Vranizan KM, Wood PD (1994). The effects of weight loss by exercise or by dieting on plasma high- density lipoprotein (HDL) levels in men with low, intermediate, and normal-to-high HDL at baseline. Metabolism.

[26] Borghouts LB, Keizer HA (2000). Exercise and insulin sensitivity: a review. Int J Sports Med.

[27] Jurimae J, Purge P, Jurimae T (2005). Adiponectin is altered after maximal exercise in highly trained male rowers. Eur J Appl Physiol.

[28] Jamurtas AZ, Theocharis V, Koukoulis G, Stakias N, Fatouros IG, Kouretas D (2006). The effects of acute exercise on serum adiponectin and resistin levels and their relation to insulin sensitivity in overweight males. Eur J Appl Physiol.

[29] Tan BK, Heutling D, Chen J, Farhatullah S, Adya R, Keay SD (2008). Metformin decreases the adipokine vaspin in overweight women with polycystic ovary syndrome concomitant with improvement in insulin sensitivity and a decrease in insulin resistance. Diabetes.

[30] Perseghin G, Price TB, Petersen KF, Roden M, Cline GW, Gerow K (1996). Increased glucose transport-phosphorylation and muscle glycogen synthesis after exercise training in insulin-resistant subjects. N Engl J Med.

